# Dirac-harmonic maps with potential

**DOI:** 10.1007/s11005-022-01558-7

**Published:** 2022-07-02

**Authors:** Volker Branding

**Affiliations:** Faculty of Mathematics, University of Vienna, Oskar-Morgenstern-Platz 1, 1090 Vienna, Austria

**Keywords:** Dirac-harmonic map with potential, Regularity, Second variation, 53C27, 58E20, 35J61

## Abstract

We study the influence of an additional scalar potential on various geometric and analytic properties of Dirac-harmonic maps. We will create a mathematical wish list of the possible benefits from inducing the potential term and point out that the latter cannot be achieved in general. Finally, we focus on several potentials that are motivated from supersymmetric quantum field theory.

## Introduction and results

The supersymmetric nonlinear sigma model has received a lot of interest in modern quantum field theory, in particular in string theory, over the past decades. At the heart of this model is an action functional whose precise structure is fixed by the invariance under various symmetry operations.

In the physics literature, the model is most often formulated in the language of supergeometry which is necessary to obtain the invariance of the action functional under supersymmetry transformations. For the physics background of the model, we refer to [1] and [18,  Chapter 3.4].

However, if one drops the invariance under supersymmetry transformations the resulting action functional can be investigated within the framework of geometric variational problems and many substantial results in this area of research could be achieved in the last years.

To formulate this mathematical version of the supersymmetric nonlinear sigma model, one fixes a Riemannian spin manifold $(M,g)$, a second Riemannian manifold $(N,h)$, and considers a map $\phi :M\rightarrow N$. The central ingredients in the resulting action functional are the Dirichlet energy for the map $\phi$ and the Dirac action for a spinor defined along the map $\phi$. In the physics literature, this spinor would also take values in a Grassmann algebra turning it into a non-commuting object. Although we neglect the invariance under supersymmetry transformations, the action functional is invariant under diffeomorphisms on the domain and for a two-dimensional domain it is also invariant under conformal transformations of the metric on the domain. The first invariance gives rise to the stress–energy tensor which is conserved whenever we consider a critical point of the action functional. The conformal invariance in dimension two leads to various nice properties of the critical points such as the possibility of removing isolated point singularities whenever a certain energy is finite.

The mathematical study of the supersymmetric nonlinear sigma model with standard spinors was initiated in [17] where the terminology *Dirac-harmonic maps* for the critical points was established. The Dirac-harmonic map equations consist of a semilinear second-order elliptic equation for the map $\phi$ and a linear Dirac equation, which is elliptic and of first order, for the spinor along the map.

Motivated from various variants in the physics literature, several extensions of Dirac-harmonic maps have also been studied from a mathematical point of view: Taking into account a two-form contribution in the action functional one is led to *magnetic Dirac-harmonic maps* [4], Dirac-harmonic maps to target spaces with torsion are investigated in [6]. Adding a curvature term to the action functional, which is quartic in the spinors, one obtains *Dirac-harmonic maps with curvature term*, which have been studied extensively in [5, 7, 8, 11, 14, 16, 29]. Recently, another extension of Dirac-harmonic maps receives growing interest: Here, one considers an additional field in the action functional, the so-called *gravitino* [26]. In the physics literature, the gravitino is the supersymmetric partner of the metric on the domain, meaning that both can be transformed into each other by a supersymmetry transformation.

At present, many results on the qualitative behavior of a given Dirac-harmonic map are known as, for example, the regularity of weak solutions [39].

However, it remains a challenging mathematical problem to prove a general existence result for Dirac-harmonic maps. The existence of uncoupled Dirac-harmonic maps using the Atiyah–Singer index theorem was established in [3]. The terminology uncoupled here refers to the fact that these Dirac-harmonic maps are constructed out of a given harmonic map such that all terms in the Euler–Lagrange equations for Dirac-harmonic maps vanish individually. An existence result for Dirac-harmonic maps from surfaces with boundary via the heat flow method could be achieved in [28].

Let us mention another recent approach to the existence problem for Dirac-harmonic maps. Motivated from the classical work of Sacks and Uhlenbeck [37], for harmonic maps, the so-called $\alpha$-*Dirac-harmonic maps* have been introduced and investigated in a series of articles [32–34] and some first existence results for Dirac-harmonic maps from closed surfaces could be achieved within this framework.

There are also some existence results available for the hyperbolic version of Dirac-harmonic maps, which are *Dirac-wave maps*, and their extensions. These arise if one considers a domain manifold with a Lorentzian metric. Existence results for Dirac-wave maps from two-dimensional Minkowski space have been obtained in [13, 21]. The Cauchy problem for Dirac-wave maps with curvature term on expanding spacetimes has been successfully studied in [15].

Besides the aforementioned existence results, several Liouville-type results have also been established [11, 12, 14, 16]. These provide criteria under which a Dirac-harmonic map must be trivial, that is the map part maps to a point and the spinor vanishes identically.

For the current state of research on the mathematical aspects of the supersymmetric nonlinear sigma model, we refer to the recent survey article [27].

In the physics literature, there exists a version of the supersymmetric nonlinear sigma model coupled to a scalar potential. This potential has to be chosen in such a way that it respects the invariance of the action functional under supersymmetry transformations. This particular potential was introduced in [2]; see also the discussion in [18,  Theorem 3.82].

Besides the mathematical results that build on the nonlinear supersymmetric sigma model, let us also recall several results on harmonic maps coupled to a scalar potential that were studied in the mathematics literature. Harmonic maps coupled to a scalar potential were introduced in [19], and the corresponding heat flow was studied in [20]. For the current status of research on harmonic maps with potential, we refer to the introduction of [9], the regularity of weak harmonic maps with (smooth) potential from surfaces was recently established in [10,  Theorem 1.1].

In this article, we will investigate the action functional for Dirac-harmonic maps coupled to an arbitrary scalar potential. Initially, one could hope that it is favorable to also include such a potential term in the action functional. Many of the difficulties in the analysis of Dirac-harmonic maps have their origin in the fact that the corresponding action functional is unbounded from below. An additional potential term can in principle be used to repair this flaw.

Let us now describe the mathematical setup that we employ in more detail. We assume that $(M,g)$ is a closed Riemannian spin manifold with spinor bundle $\Sigma M$; for more details about spin geometry, see the book [35]. Moreover, let $(N,h)$ be a second closed Riemannian manifold. Let $\phi :M\rightarrow N$ be a map; integrating the square of its differential $\mathrm{d}\phi \in \Gamma (T^*M\otimes \phi ^*TN)$ leads to the usual Dirichlet energy. Together with the pullback bundle $\phi ^*TN$, we consider the twisted bundle $\Sigma M\otimes \phi ^*TN$. The induced connection on this bundle will be denoted by $\tilde{\nabla }$. Moreover, we have an induced Hermitian scalar product on $\Sigma M\otimes \phi ^*TN$ of which we will always take the real part. Sections $\psi \in \Gamma (\Sigma M\otimes \phi ^*TN)$ in this bundle are called *vector spinors*. The natural operator acting on vector spinors is the twisted Dirac operator, denoted by . More precisely, the twisted Dirac operator is given by , where $\{e_i\},i=1,\ldots ,m=\dim M$ is an orthonormal basis of $TM$ and $\cdot$ denotes Clifford multiplication. It is an elliptic, first-order operator, which is self-adjoint with respect to the $L^2$-norm. We are using the Einstein summation convention, that is we sum over repeated indices. Whenever choosing indices, we will use Latin letters for indices related to $M$ and Greek letters for indices on $N$.

Recall that Clifford multiplication is skew-symmetric, namely

$$\begin{aligned} \langle \chi ,X\cdot \xi \rangle _{\Sigma M}=-\langle X\cdot \chi ,\xi \rangle _{\Sigma M} \end{aligned}$$

for all $\chi ,\xi \in \Gamma (\Sigma M)$ and all $X\in TM$. In addition, the Clifford relations

$$\begin{aligned} X\cdot Y+Y\cdot X=-2g(X,Y) \end{aligned}$$

hold for all $X,Y\in TM$.

In terms of local coordinates $y^\alpha$ on $N$, the vector spinor $\psi$ can be expanded as $\psi =\psi ^\alpha \otimes \frac{\partial }{\partial y^\alpha }$, and thus the twisted Dirac operator  is locally given byHere,  denotes the standard Dirac operator and $\Gamma ^\alpha _{\beta \gamma }$ are the Christoffel symbols of the manifold $N$.

In order to define Dirac-harmonic maps with potential, assume that $V:\Sigma M\otimes \phi ^*TN\times N\rightarrow \mathbb {R}$ is a function which we assume to be smooth in the following.

The central object in this article is the action functional1.1where we use the subscript $P$ to highlight the presence of the potential $V(\phi ,\psi )$.

Before we turn to the mathematical analysis of the action functional (1.1), let us give a mathematical wish list of what the potential $V(\phi ,\psi )$ could achieve:

1. The potential $V(\phi ,\psi )$ removes the indefinite character of the action functional (1.1) such that $S_P(\phi ,\psi )$ is now bounded from below. Moreover, the potential can help in establishing an existence result for critical points of (1.1).
2. The potential also ensures that critical points of (1.1) are stable in the sense that the second variation is positive under certain geometric or analytic assumptions.
3. The regularity theory for weak solutions of the Dirac-harmonic map system (which are the critical points of (1.1) in the case of a vanishing potential) also applies to the critical points of (1.1).
4. The potential respects the invariance under diffeomorphisms on the domain and also under conformal transformations in the case of a two-dimensional domain manifold.

Even without going into the mathematical details, one should expect that there cannot be a potential $V(\phi ,\psi )$ that satisfies all items of the above wish list. For example, if we have a potential that is able to suppress the negative eigenvalues of the twisted Dirac operator , then we cannot expect it to be integrable in the Sobolev spaces that are used in the regularity analysis of weak Dirac-harmonic maps.

The inclusion of a fine-tuned potential term turned out to be very helpful in order to derive existence results for perturbed versions of Dirac-harmonic maps as was done in [23–25]. However, one still needs highly sophisticated techniques from the calculus of variation in order to achieve such results.

This article is organized as follows: In Sect. 2, we calculate the first and the second variation of (1.1). The third section studies the possible benefits of the scalar potential $V(\phi ,\psi )$. In particular, we investigate if it can help to ensure the positivity of the action functional (1.1) and its second variation. Moreover, we make some comments on the importance of the second variation of an action functional for quantum field theory. Section 4 focuses on the regularity of weak Dirac-harmonic maps with potential.

The last section discusses various explicit potentials, both from the physics literature and inspired from the mathematical wish list above, and their possible benefit for the mathematical analysis.

## Variational formulas

In this section, we derive several variational formulas for Dirac-harmonic maps with potential. In the following, $\epsilon$ will always denote a small positive real number.

### First variation

Let us briefly discuss the first variation of the action functional (1.1).

#### Proposition 2.1

The critical points of the action functional (1.1) are given by2.12.2where $\tau (\phi )$ is the tension field of the map $\phi$, $V_{\phi }(\phi ,\psi )$ is the functional derivative of the potential $V(\phi ,\psi )$ with respect to the map $\phi$ and $V_{\psi }(\phi ,\psi )$ represents the functional derivative of the potential $V(\phi ,\psi )$ with respect to the spinor $\psi$.

#### Proof

We consider a variation of the pair $(\phi ,\psi )$, given by $\phi _t:(-\epsilon ,\epsilon )\times M\rightarrow N$ and $\psi _t:(-\epsilon ,\epsilon )\times M\rightarrow \Sigma M\otimes \phi _t^*TN$ that satisfies $\frac{\partial \phi _t}{\partial t}\big |_{t=0}=\eta$ and $\frac{\tilde{\nabla }\psi _t}{\partial t}\big |_{t=0}=\xi$.

The following variational formulas are well known; see for example [26,  Section 4]:In order to obtain the variation of the potential $V(\phi ,\psi )$, we employ the chain rule and find

$$\begin{aligned} \frac{\hbox {d}}{\hbox {d}t}\big |_{t=0} V(\phi _t,\psi _t)&=\langle V_{\phi _t}(\phi _t,\psi _t),\mathrm{d}\phi _t(\partial _t)\rangle |_{t=0} +\langle V_{\psi _t}(\phi _t,\psi _t),\frac{\tilde{\nabla }\psi _t}{\partial t}\rangle |_{t=0}\\&=\langle V_\phi (\phi ,\psi ),\eta \rangle +\langle V_{\psi }(\phi ,\psi ),\xi \rangle . \end{aligned}$$

The claim follows from combining the different equations. $\square$

Let us give the local version of the Euler–Lagrange equations (2.1), (2.2). To this end, let $(U,x^i)$ be a local chart on $M$ and $(V,y^\alpha )$ be a local chart on $N$ such that $\phi (U)\subset V$. Then, the system (2.1), (2.2) acquires the form

#### Remark 2.2

The functional derivative of $V(\phi ,\psi )$ with respect to $\psi$ in (2.2) can be defined as follows: Consider a variation of the vector spinor $\psi$ that is $\psi _t:(-\epsilon ,\epsilon )\times M\rightarrow \Sigma M\otimes \phi ^*TN$ that satisfies $\frac{\tilde{\nabla }\psi _t}{\partial t}\big |_{t=0}=\xi$ while keeping the map $\phi$ fixed. Then, $V_\psi (\phi ,\psi )\in \Gamma (\Sigma M\otimes \phi ^*TN)$ is defined as follows:

2.3 $$\begin{aligned} \langle V_\psi (\phi ,\psi ),\xi \rangle :=\frac{\hbox {d}}{\hbox {d}t}\big |_{t=0}V(\phi ,\psi _t). \end{aligned}$$

### Second variation

In this section, we will calculate the second variation of the action functional (1.1). In general, one should of course expect the second variation of (1.1) to be indefinite due to the presence of the Dirac term. Again, we make us of a variation of the pair $(\phi ,\psi )$, given by $\phi _t:(-\epsilon ,\epsilon )\times M\rightarrow N$ and $\psi _t:(-\epsilon ,\epsilon )\times M\rightarrow \Sigma M\otimes \phi _t^*TN$ satisfying

$$\begin{aligned} \frac{\partial \phi _t}{\partial t}\big |_{t=0}=\eta ,\qquad \frac{\tilde{\nabla }\psi _t}{\partial t}\big |_{t=0}=\xi . \end{aligned}$$

Using the first variation (2.1), (2.2), we obtainIn order to obtain the formula for the second variation of (1.1), we have to differentiate the above equation with respect to $t$ once more and evaluate it at $t=0$.

It is well known that

$$\begin{aligned} \frac{\hbox {d}}{\hbox {d}t}\big |_{t=0}\int _M\langle \mathrm{d}\phi _t(\partial _t),\tau (\phi _t)\rangle \text {dvol}_g=&\int _M\left\langle \frac{\partial ^2\phi _t}{\partial t^2},\tau (\phi _t)\right\rangle \text {dvol}_g\big |_{t=0} \\&+\int _M(-|\nabla \eta |^2+\langle R^N(\eta ,\mathrm{d}\phi )\mathrm{d}\phi ,\eta \rangle )\text {dvol}_g. \end{aligned}$$

Moreover, we find that

$$\begin{aligned} \frac{\hbox {d}}{\hbox {d}t}\big |_{t=0}\left( \frac{1}{2}R^N(\psi _t,e_i\cdot \psi _t)\mathrm{d}\phi _t(e_i)\right) =&\frac{1}{2}(\nabla _\eta R^N)(\psi ,e_i\cdot \psi )\mathrm{d}\phi (e_i) \\&+R^N(\xi ,e_i\cdot \psi )\mathrm{d}\phi (e_i)+\frac{1}{2}R^N(\psi ,e_i\cdot \psi )\nabla _{e_i}\eta \end{aligned}$$

leading to

$$\begin{aligned} \frac{\hbox {d}}{\hbox {d}t}\big |_{t=0}\int _M&\langle \mathrm{d}\phi _t(\partial _t), \frac{1}{2}R^N(\psi _t,e_i\cdot \psi _t)\mathrm{d}\phi _t(e_i)\rangle \text {dvol}_g\\ =&\int _M\bigg (\bigg \langle \frac{\partial ^2\phi _t}{\partial t^2}, \frac{1}{2}R^N(\psi _t,e_i\cdot \psi _t)\mathrm{d}\phi _t(e_i)\bigg \rangle \text {dvol}_g\big |_{t=0}\\&+\frac{1}{2}\langle \eta ,(\nabla _\eta R^N)(\psi ,e_i\cdot \psi )\mathrm{d}\phi (e_i)\rangle \\&+\langle \eta ,R^N(\xi ,e_i\cdot \psi )\mathrm{d}\phi (e_i)\rangle +\frac{1}{2}\langle \eta ,R^N(\psi ,e_i\cdot \psi )\nabla _{e_i}\eta \rangle \bigg )\text {dvol}_g. \end{aligned}$$

The second variation of the Dirac action can be calculated asRegarding the potential term $V(\phi ,\psi )$, we find by using the chain rule

$$\begin{aligned} \frac{\hbox {d}^2}{\hbox {d}t^2}\big |_{t=0}V(\phi _t,\psi _t)&=\frac{\hbox {d}}{\hbox {d}t}\big |_{t=0}\big (\langle V_{\phi _t}(\phi _t,\psi _t),\mathrm{d}\phi _t(\partial _t)\rangle +\langle V_{\psi _t}(\phi _t,\psi _t),\frac{\tilde{\nabla }\psi _t}{\partial t}\rangle \big )\\&=\left\langle \frac{\partial ^2\phi _t}{\partial t^2},V_{\phi _t}(\phi _t,\psi _t)\right\rangle \big |_{t=0} +\left\langle \frac{\tilde{\nabla }^2\psi _t}{\partial t^2},V_\psi (\phi _t,\psi _t)\right\rangle \big |_{t=0} \\&\quad +\iota (\eta ,\eta )\big (V_{\phi \phi }(\phi ,\psi )\big )+\iota (\xi ,\xi )\big (V_{\psi \psi }(\phi ,\psi )\big )\\&\quad +2\iota (\eta ,\xi )\big (V_{\phi \psi }(\phi ,\psi )\big ). \end{aligned}$$

Here, and in the following, the symbol $\iota$ is used to either denote the insertion of a vector field $\eta$ or a vector spinor $\xi$ into the corresponding functional derivative of the potential.

Combining the previous steps, we get

#### Theorem 2.3

Let $(\phi ,\psi )$ be a smooth solution of the system (2.1), (2.2). Then, the second variation of the action functional (1.1) is given by2.4

#### Proof

This follows from the previous equations and the fact that $(\phi ,\psi )$ is a smooth solution of the system (2.1), (2.2). $\square$

Formally, we can interpret the second variation of (1.1) in the following way:

$$\begin{aligned} {{\text {Hess}}~}\big (S_P(\phi ,\psi )\big )(\eta ,\xi )=&\int _M\langle I(\eta ,\xi ),(\eta ,\xi )\rangle \text {dvol}_g, \end{aligned}$$

where $I(\phi ,\psi ):\Gamma (\phi ^*TN)\times \Gamma (\Sigma M\otimes \phi ^*TN)\rightarrow \Gamma (\phi ^*TN)\times \Gamma (\Sigma M\otimes \phi ^*TN)$ is a semilinear elliptic differential operator of order 2 that generalizes the well-studied *Jacobi operator*.

We can define the associated Jacobi operator of (1.1) as follows:2.5Here, we have $\eta \in \Gamma (\phi ^*TN), \xi \in \Gamma (\Sigma M\otimes \phi ^*TN)$ and $\Delta$ is the rough Laplacian on the vector bundle $\phi ^*TN$. Note that the Jacobi operator cannot be defined in a unique fashion as we can assign $\eta$ and $\xi$ contained in the term $\langle \eta ,R^N(\xi ,e_i\cdot \psi )\mathrm{d}\phi (e_i)\rangle$ to either the equation for $\eta$ or the equation for $\xi$.

## Positivity aspects and the question of stability

The initial mathematical motivation to study Dirac-harmonic maps coupled to a scalar potential was the hope that a suitable potential may cure at least some of the analytic difficulties that arise in the analysis of Dirac-harmonic maps, in particular the problems arising from the unboundedness of the action functional. In this section, we will analyze this idea in some more detail.

First, we will have a look at the action functional (1.1). If we assume that $M$ is compact, then we know from general spectral theory that the twisted Dirac operator  has a discrete spectrum consisting of an unbounded discrete sequence of positive and negative eigenvalues. If we expand a vector spinor $\psi$ with respect to a basis of eigenspinors $\psi _I$ as

$$\begin{aligned} \psi :=\sum _{I=-\infty }^\infty \alpha _I\psi _I,\qquad \alpha _I\in \mathbb {C}, \end{aligned}$$

the Dirac term in the action functional (1.1) acquires the formHere, we used that , where $\lambda _J$ represents the Jth eigenvalue of the twisted Dirac operator .

Consequently, if we want to obtain a lower bound for the action functional (1.1), we need to require that

3.1 $$\begin{aligned} \sum _{J=-\infty }^\infty |\alpha _J|^2\lambda _J-2\int _MV(\phi ,\psi )\text {dvol}_g\ge 0. \end{aligned}$$

We can conclude that the potential $V(\phi ,\psi )$ needs to be able to approach infinity in the same fashion as the spectrum of the twisted Dirac operator  does. However, if we want to allow the potential $V(\phi ,\psi )$ to be smooth and to choose a compact target manifold $N$, then it will not be able to compensate the lowest eigenvalue of the Dirac operator. On the other hand, if we choose a non-compact target manifold $N$, we might encounter additional difficulties in dealing with the analytic aspects of the critical points of (1.1).

### Positivity of the second variation

In the geometric calculus of variation, the second variation of a given action functional encodes important information on the stability of a critical point. In the case of harmonic maps, it is well known that the second variation of the Dirichlet energy is positive when the target manifold has negative curvature such that critical points are stable then [22].

In the case of Dirac-harmonic maps (with or without additional potential), one again cannot expect the second variation of the action functional to be positive in general due to the presence of the Dirac term. Let us again derive a condition on the potential $V(\phi ,\psi )$ that would be sufficient to ensure the positivity of the second variation. To this end, we estimate the second variation (2.4) as follows:Here, $c_i,i=1,\ldots ,6$ are positive constants that only depend on the geometry of $N$.

In the analysis of Dirac-harmonic maps and their variants, the following energy often turns out to be crucial

$$\begin{aligned} e(\phi ,\psi )=|\mathrm{d}\phi |^2+|\psi |^4. \end{aligned}$$

The second variation of (1.1) would be positive if the following conditions hold:

1. The Hessian of the potential with respect to the map $\phi$ needs to satisfy
  3.2 $$\begin{aligned} -\iota (\eta ,\eta )\big (V_{\phi \phi }(\phi ,\psi )\big )\ge C|\eta |^2e(\phi ,\psi ) \end{aligned}$$
  for some positive constant $C$. Again, we realize that we would need to choose a non-compact target manifold in order for the Hessian of the potential to be negative globally.
2. In addition, we need to require the positivity of the following expression: 3.3

The analysis carried out in this section strongly suggests that it will not be possible to find a “suitable” potential $V(\phi ,\psi )$ that can ensure the positivity of the second variation of the action functional (1.1) and that also has a reasonable application in quantum field theory.

### Some remarks on the notion of stability and quantum field theory

Let us also make a remark on why the stability of a critical point of an action functional, within the framework of the geometric calculus of variations, could have important implications for calculations in quantum field theory.

In the context of geometric variational problems, the second variation of an action functional gives important information on the stability of a critical point. Roughly speaking, if the second variation of an action functional is positive, one knows that the corresponding solution is stable. More precisely, this means that the critical point is isolated in the space of all solutions and there does not exist a solution “nearby.” The standard example to illustrate this fact is geodesics in hyperbolic space (which are isolated) in contrast to geodesics on the sphere (in which case we can always find a geodesic nearby). This fact also holds true for higher-dimensional domains: Harmonic maps to targets with negative curvature are stable [22], whereas harmonic maps to spheres will be unstable in general due to a classic result of Leung [36].

In quantum field theory, several notions of stability exist; however, they are usually not connected to the second variation of the action functional. Nevertheless, we would like to point out that also for the calculations in physics, in particular in the context of quantum field theory on an Euclidean space, an understanding of the second variation should be of great importance.

Assuming that we have a critical point of an action functional in quantum field theory (which corresponds to a classical solution in physics), one would like to quantize this solution. Let us suppose that one can perform this quantization procedure in a rigorous mathematical manner, then one can think of it as calculating small corrections to the classical solution. However, if the classical solution is not stable, any small perturbation of it might change the classical solution to a nearby solution. This might have the effect that one effectively does not calculate the correction to the classical solution one is looking for but just switches to a classical solution nearby.

Note that these considerations are only valid if one chooses a Riemannian domain as action functionals on Lorentzian manifolds are not positive definite in general, and consequently it does not make much sense to investigate the second variation in this case.

The *SO(n)*-nonlinear sigma model is a well-studied model in quantum field theory. In the physics literature, this model is considered both on Riemannian and on Lorentzian manifolds and one often employs the method of Wick rotation to switch between both of them. It should of course be noted that the method of Wick rotation is not a rigorous mathematical operation in general. In mathematical terms, the *SO(n)*-nonlinear sigma model corresponds to harmonic/wave maps with a spherical target of dimension $n$.

Hence, in the case of a Riemannian domain, our above remarks on the instability of harmonic maps to spheres can be applied to the *SO(n)*-nonlinear sigma model. This model is often used to describe the low-energy behavior of certain particles, the so-called *pions*; see [40,  Chapter 19] and references therein for more details on the physics background. However, one observes that pions are unstable in the sense that they decay into other particles. This might very well be a consequence of the fact that the second variation of the corresponding action functional is not positive. Consequently, it may not be necessary to perform any kind of quantization procedure as is done in physics to explain the instability of pions.

Moreover, as we have seen the critical points of the supersymmetric version of the nonlinear sigma model will be unstable as well in general even if we take into account an additional scalar potential.

## Regularity of weak solutions

In this section, we investigate the regularity of weak solution of the system (2.1), (2.2). Our analysis is very similar to the regularity analysis of the nonlinear sigma model coupled to a gravitino which was carried out in [31,  Section 2] and to the one for Dirac-harmonic maps with curvature term [5,  Section 4.1] for $\dim =2$ and [29] for the higher-dimensional case. The methods used to control the gravitino of [31] can be modified such that they also apply to our setup.

Let us emphasize again that we are assuming the potential $V(\phi ,\psi )$ to be smooth. The case of a non-smooth potential can be treated with the help of the methods developed in [30] for the nonlinear sigma model coupled to a non-smooth gravitino.

Since we assume to have a compact target manifold, the contributions of the potential $V(\phi ,\psi )$ that do not depend on $\psi$ are always bounded by a constant.

Motivated by the structure of various potentials that appear in quantum field theory, we will first focus on a potential of the following form:

4.1 $$\begin{aligned} V(\phi ,\psi )=H(\phi )+G_{\alpha _1\alpha _2\ldots \alpha _s}(\phi )\langle \psi ^{\alpha _1}, \psi ^{\alpha _2}\rangle _{\Sigma M}\times \ldots \times \langle \psi ^{\alpha _{s-1}},\psi ^{\alpha _{s}}\rangle _{\Sigma M}. \end{aligned}$$

Here, $H(\phi ), G(\phi )$ are smooth functions on $N$ and the indices $0\le \alpha _i\le \cdots \le \alpha _s$ are used to contract the spinors in the potential, where $s$ needs to be an even number.

Hence, for the class of potentials from above the following growth assumption holds:

4.2 $$\begin{aligned} |V(\phi ,\psi )|\le C_1+C_2|\psi |^s, \end{aligned}$$

for some positive constants $C_1,C_2$. In particular, we have

4.3 $$\begin{aligned} |V_\phi (\phi ,\psi )|&\le C_3+C_4|\psi |^s,\nonumber \\ |V_\psi (\phi ,\psi )|&\le C_5|\psi |^{s-1} \end{aligned}$$

for positive constants $C_i,i=3,\ldots ,5$.

First, we define a weak solution of the system (2.1), (2.2).

### Definition 4.1

Suppose that the potential $V(\phi ,\psi )$ is of the structure (4.1). A weak solution $(\phi ,\psi )$ of the system (2.1), (2.2) is a critical point of the action functional (1.1) in the Sobolev space

$$\begin{aligned} \chi _t(M,N):=W^{1,2}(M,N)\times W^{1,\frac{4}{3}}(\Sigma M\otimes \phi ^*TN)\times L^t(\Sigma M\otimes \phi ^*TN), \end{aligned}$$

where $t=4$ if $0<s\le 4$ and $t=s$ if $s>4$.

### Remark 4.2

The way we have defined the space $\chi _t(M,N)$ is inspired from the analysis of Dirac-harmonic maps which corresponds to solutions of (2.1), (2.2) with $V(\phi ,\psi )=0$. This fits naturally to how we have defined weak solutions above as the action functional (1.1) is finite whenever $(\phi ,\psi )\in \chi _t(M,N)$. For a potential with stronger growth as in (4.2), we will have to adjust the space $\chi _t(M,N)$ in such a way that (1.1) is finite.

In order to state the main regularity result, we recall the following:

### Definition 4.3

For a given open subset $D\subset \mathbb {R}^m$ and $1\le p<\infty$, $0<\lambda \le m$, the Morrey space $M^{p,\lambda }(D)$ is defined as follows:

$$\begin{aligned} M^{p,\lambda }(D):=\left\{ f\in L^p(D)\mid \Vert f\Vert ^p_{M^{p,\lambda }(D)}:=\sup _{B_r\subset D}\{r^{\lambda -m} \int _{B_r}|f|^p(y)dy\}<\infty \right\} . \end{aligned}$$

Note that $$M^{p,m}(D)=L^p(D)$$.

The following smallness condition will be important in the formulation of our main regularity result:

4.4 $$\begin{aligned} \Vert \mathrm{d}\phi \Vert _{M^{2,2}(U)}+\Vert \psi \Vert _{M^{4,2}(U)}\le \epsilon \end{aligned}$$

Here, $\epsilon >0$ is a small number depending on $(M,g), (N,h)$ and $U$ an open subset of $M$.

We will prove the following regularity result for weak solutions of the Dirac-harmonic map with potential system:

### Theorem 4.4

Let $m\ge 2$ and suppose that the potential $V(\phi ,\psi )$ is smooth and has the structure (4.1). We need to make the following case distinction:

1. If $1<s\le 4$, suppose that (4.4) holds.
2. If $s>4$, suppose that (4.4) holds and also $\psi \in {M^{2,2s-2}(U)}$.

Then, a weak solution $(\phi ,\psi )\in \chi _t(M,N)$ is smooth in $U$, where $U$ is an open subset of $M$.

### Remark 4.5

1. For $\dim M=2$, the smallness condition (4.4) can easily be achieved. In this case, it reads $\Vert \mathrm{d}\phi \Vert _{L^2(U)}+\Vert \psi \Vert _{L^4(U)}\le \epsilon$ and can be satisfied by choosing $U$ sufficiently small.
2. Due to the Sobolev embedding theorem, we have $\Vert \psi \Vert _{L^\frac{m}{\frac{3}{4}m-1}(U)}\le C\Vert \nabla \psi \Vert _{L^\frac{4}{3}(U)}$ such that for $\dim M=2$ we do not have to require that $\psi \in L^4(\Sigma M\otimes \phi ^*TN)$ in the definition of the space $\chi _t(M,N)$ if $0<s\le 4$. The Sobolev embedding theorem does not help in the case of $m\ge 3$ as we have $\frac{m}{\frac{3}{4}m-1}<4$.
3. Note that Theorem 4.4 and its proof can easily be modified such that they also hold for non-integer values of $s$.

In order to prove Theorem 4.4, it is favorable to apply the embedding theorem of Nash and to isometrically embed the target manifold $N$ into some $\mathbb {R}^q$ of sufficiently large dimension $q$. We denote this isometric embedding by $\iota :N\rightarrow \mathbb {R}^q$ which we assume to be smooth. Moreover, let $\phi ':=\iota \circ \phi :M\rightarrow \mathbb {R}^q$ and $\psi '=\mathrm{d}\iota (\psi )$ be the corresponding pushforward of the vector spinor. As $\psi \in \Gamma (\Sigma M\otimes \phi ^*TN)$ behaves like a tangent vector on $N$, we have to use the differential of $\iota$ to push it to the ambient space $\mathbb {R}^q$; hence, we have $\psi '=\mathrm{d}\iota (\psi )\in \Gamma (\Sigma M\otimes \mathbb {R}^q)$.

Set $n:=\dim N$ and let $u^\alpha ,1\le \alpha \le q$ be global coordinates on the ambient space $\mathbb {R}^q$. Moreover, let $\nu _\theta ,\theta =n+1,\ldots ,q$ be an orthonormal frame of the submanifold $\iota (\phi )$. In this setup, $\phi '$ becomes a vector-valued function and $\psi '$ can be thought of as a vector of standard spinors $\psi '^\alpha \in \Gamma (\Sigma M),1\le \alpha \le q$ that are constrained as follows:

$$\begin{aligned} \sum _{\alpha }\nu ^\alpha _\theta \psi '^\alpha =0,\qquad n+1\le \theta \le q. \end{aligned}$$

This allows us to give the extrinsic version of the Euler–Lagrange equations (2.1), (2.2).

In the following, we will omit the $'$ and again write $\phi ,\psi$ for the extrinsic version of the Euler–Lagrange equations to shorten the notation.

### Lemma 4.6

The extrinsic version of the Euler–Lagrange equations (2.1), (2.2) for $\phi :M\rightarrow \mathbb {R}^q$ and $\psi \in \Gamma (\Sigma M\otimes \mathbb {R}^q)$ is given by the system

4.5 $$\begin{aligned} \Delta \phi ^\alpha&=(\omega _{i}^{\alpha \beta }+F_i^{\alpha \beta }) \frac{\partial \phi ^\beta }{\partial x^i}-(V_\phi (\phi ,\psi ))^\alpha , \end{aligned}$$

4.6where $$1\le \alpha \le q$$ and

$$\begin{aligned} \omega _{i}^{\alpha \beta }&=\frac{\partial \phi ^\gamma }{\partial x^i}\frac{\partial \nu _l^\beta }{\partial u^\gamma }\nu _l^\alpha -\frac{\partial \phi ^\gamma }{\partial x^i}\frac{\partial \nu _l^\alpha }{\partial u^\gamma }\nu _l^\beta =-\omega _{i}^{\alpha \beta },\\ F_i^{\alpha \beta }&=\langle \psi ^\gamma ,e_i\cdot \psi ^\delta \rangle \bigg (\big (\frac{\partial \nu _l}{\partial u^\delta }\big )^{\top ,\beta }\big (\frac{\partial \nu _l}{\partial u^\gamma }\big )^{\top ,\alpha } -\big (\frac{\partial \nu _l}{\partial u^\delta }\big )^{\top ,\alpha }\big (\frac{\partial \nu _l}{\partial u^\gamma }\big )^{\top ,\beta } \bigg ) =-F_i^{\beta \alpha }. \end{aligned}$$

Here, $\top$ denotes the projection map $\top :\mathbb {R}^q\rightarrow TN$.

### Proof

We will only treat the terms involving the potential $V(\phi ,\psi )$; for the remaining terms, see the detailed discussion in [26,  Section 4.3].

Suppose that ${\tilde{V}}_\phi (\phi ',\psi ')$ is an extension of $V_\phi (\phi ,\psi )$ to the ambient space $\mathbb {R}^q$. Then, using the projection $\top$ to the tangent space $T_yN$ at the point $y\in N$ we can set

$$\begin{aligned} ({\tilde{V}}_\phi (\phi ',\psi '))^\top =V_\phi (\phi ,\psi ). \end{aligned}$$

We can also extend $V_\psi (\phi ,\psi )$ to the ambient space, denote this extension by ${\tilde{V}}_\psi (\phi ,\psi )$. By the same argument as before we set

$$\begin{aligned} ({\tilde{V}}_\psi (\phi ',\psi '))^\top =V_\psi (\phi ,\psi ) \end{aligned}$$

completing the proof. $\square$

Abbreviating

$$\begin{aligned} \Omega ^{\alpha \beta }_i=\omega _{i}^{\alpha \beta }+F_i^{\alpha \beta }, \qquad A_{\alpha \beta }=-\nabla \phi ^\delta \frac{\partial \nu _l^\beta }{\partial u^\delta }\nu _l^\alpha \end{aligned}$$

the extrinsic version of the Euler–Lagrange equations (4.5), (4.6) can be written in the compact form4.7with the important feature that $\Omega$ is antisymmetric, that is $\Omega ^{\alpha \beta }=-\Omega ^{\beta \alpha }$. Note that we have $|\Omega |^2\le C(|\mathrm{d}\phi |^2+|\psi |^4)$ and $|A|\le C|\mathrm{d}\phi |$.

At this point, we are ready to apply tools from elliptic regularity that will help to improve the integrability of the system (4.7).

On the one hand, we will make use of the following Lemma, which was proven in [26,  Lemma 6.1]:

### Lemma 4.7

Let $B_1\subset \mathbb {R}^m$, $m\ge 2$ and let $4<p<\infty$. Consider a weak solution $\varphi \in M^{4,2}(B_1,\mathbb {R}^L\otimes \mathbb {R}^q)$ of4.8where $A\in M^{2,2}(B_1,\mathfrak {gl}(L,\mathbb {R})\otimes \mathfrak {gl}(q,\mathbb {R}))$ and $B\in M^{2,2}(B_1,\mathbb {R}^L\otimes \mathbb {R}^q)$. Then, there exists $\epsilon _0=\epsilon _0(m,p)>0$ such that if

$$\begin{aligned} \Vert A\Vert _{M^{2,2}(B_1)}\le \epsilon _0, \end{aligned}$$

we have $\varphi \in L^p_{loc}(B_1)$. Moreover, for any $U\subset B_1$ there holds the estimate

4.9 $$\begin{aligned} \Vert \varphi \Vert _{L^p(U)}\le C(m,p,U)(\Vert \varphi \Vert _{M^{4,2}(B_1)}+\Vert B\Vert _{M^{2,2}(B_1)}) \end{aligned}$$

for some $$C(m,p,U)>0$$.

On the other hand, in order to improve the integrability of the map $\phi$, we employ the following regularity result, which was obtained in [38,  Theorem 1.2]:

### Theorem 4.8

Let $B_1\subset \mathbb {R}^m$, $m\ge 2$ and let $u\in W^{1,2}(B_1,\mathbb {R}^q)$ with $\nabla u\in M^{2,2}(B_1,\mathbb {R}^m\otimes \mathbb {R}^q)$, $\Omega \in M^{2,2}(B_1,\mathfrak {so}(q)\otimes \mathbb {R}^m)$ and $f\in L^p(B_1)$ for $\frac{m}{2}<p<m$, be a weak solution of

$$\begin{aligned} -\Delta u=\Omega \cdot \nabla u+f. \end{aligned}$$

Then, for any $U\subset B_1$ there exists $\epsilon _1=\epsilon _1(m,p,q,U)$ such that whenever $\Vert \Omega \Vert _{M^{2,2}}\le \epsilon _1$ the following estimate holds:

4.10 $$\begin{aligned} \Vert \nabla ^2u\Vert _{M^{\frac{2p}{m},2}(U)}+\Vert \nabla u\Vert _{M^{\frac{2p}{m-p},2}(U)}\le C(\Vert u\Vert _{L^1(B_1)}+\Vert f\Vert _{L^p(B_1)}). \end{aligned}$$

Note that in [38] a different convention in the definition of Morrey spaces is used as in this article.

### Remark 4.9

We can think of Lemma 4.7 as providing a regularity result for Dirac equations coupled to a potential, whereas Theorem 4.8 constitutes a regularity result for Laplace-type equations allowing a gradient term on the right-hand side with an antisymmetric structure and also a potential term. While both results make use of the ellipticity of the Dirac and the Laplace operator, it becomes clear that the smoothing effects of the Laplacian are stronger: In Lemma 4.7, we need to demand that the potential lies in some Morrey space, whereas in Theorem 4.8 it is enough that the potential is integrable in $L^p$ for specific values of $p$.

We are now ready to give the proof of Theorem 4.4.

### Proof of Theorem 4.4

First, we will improve the regularity of the spinor by making use of Lemma 4.7. Thus, we use a local trivialization of $\Sigma M$ over $B_1$ and make the following case distinction:

1. If $1<s<4$, the only case to be considered is $s=2$. Then, we apply Lemma 4.7 with $|B|\le C|\psi |$. Note that $\Vert \psi \Vert _{M^{2,2}(U)}\le C\Vert \psi \Vert _{M^{4,2}(U)}\le C\epsilon$ due to the Hölder inequality in Morrey spaces.
2. If $s=4$, we can write the equation for the spinor (4.6) in the form  where $|A|\le C(|\mathrm{d}\phi |+|\psi |^2)$ such that we can apply Lemma 4.7 with $B=0$.
3. If $s>4$, we again apply Lemma 4.7 with $|B|\le C|\psi |^{s-1}$.

Due to the assumptions made in Theorem 4.4, we get from Lemma 4.7 that $\psi \in L^p_{loc}$ for any $p\in [1,\infty )$.

At this point, we are ready to improve the integrability of the map $\phi$. Using the growth condition for the potential (4.2), we can apply Theorem 4.8. Setting $p=m-\delta$, we calculate

$$\begin{aligned} \frac{2p}{m-p}=\frac{2(m-\delta )}{m-(m-\delta )}=\frac{2m}{\delta }-2 \end{aligned}$$

and choosing $\delta >0$ sufficiently small we obtain the continuity of $\phi$ from the Morrey lemma. From this point on, Theorem 4.4 follows by a standard bootstrap procedure. $\square$

Let us shortly discuss how Theorem 4.4 needs to be modified if we would consider an arbitrary potential $V(\phi ,\psi )$ that does not have the structure (4.1). In the latter case, we would need to consider the function space

$$\begin{aligned} \tilde{\chi }(M,N)&:=W^{1,2}(M,N)\times W^{1,\frac{4}{3}}(\Sigma M\otimes \phi ^*TN) \times L^4(\Sigma M\otimes \phi ^*TN)\\&\qquad \times T(\Sigma M\otimes \phi ^*TN), \end{aligned}$$

where the space $T(\Sigma M\otimes \phi ^*TN)$ needs to be chosen such that $S_P(\phi ,\psi )$ is finite for the potential under consideration.

### Theorem 4.10

Let $m\ge 2$, $U\subset M$ open and suppose that the potential $V(\phi ,\psi )$ is smooth and satisfies $\Vert V_\psi (\phi ,\psi )\Vert _{M^{2,2}}(U)<\infty$. Then, there exists $\epsilon >0$ such that if

$$\begin{aligned} \Vert \mathrm{d}\phi \Vert _{M^{2,2}(U)}+\Vert \psi \Vert _{M^{4,2}(U)}\le \epsilon , \end{aligned}$$

where $\epsilon >0$ is a small number depending on $(M,g), (N,h)$, a weak solution $(\phi ,\psi )\in \tilde{\chi }(M,N)$ is smooth in $U$.

### Proof

This again follows from Lemma 4.7 and Theorem 4.8. $\square$

## Explicit potentials from quantum field theory

In this section, we will apply our previous analysis for an arbitrary potential to some specific potentials which are studied in quantum field theory. We will shed light on the particular mathematical advantages and drawbacks of the potentials used in theoretical physics.

### Dirac-harmonic maps with curvature term

Dirac-harmonic maps with curvature term arise as critical points of (1.1) by choosing the following potential:

$$\begin{aligned} V_1(\phi ,\psi ):=\frac{1}{12}\langle R^N(\phi )(\psi ,\psi )\psi ,\psi \rangle . \end{aligned}$$

Here, the spinors are contracted in the following way:

$$\begin{aligned} \langle R^N(\psi ,\psi )\psi ,\psi \rangle =R_{\alpha \beta \gamma \delta }\langle \psi ^\alpha , \psi ^\gamma \rangle \langle \psi ^\beta ,\psi ^\delta \rangle , \end{aligned}$$

which ensures that the action functional is real-valued. This particular potential was first studied in the mathematics literature in [16].

In the physics literature, the prefactor in front of the potential is chosen such that it ensures the invariance of the action functional under supersymmetry transformations [1]. However, the mathematical analysis initiated in [16] applies to any nonzero constant in front of the curvature term.

The equations for Dirac-harmonic maps with curvature term are the following:5.15.2where $\sharp :\phi ^*T^*N\rightarrow \phi ^*TN$ represents the musical isomorphism.

For the domain being a closed surface, it was shown in [5] that a weak solution $(\phi ,\psi )\in \chi _{t}(M,N)$ of (2.1), (2.2) with $t=4$ is smooth which was later extended to higher-dimensional domains in [29].

If the domain manifold is two-dimensional, the action functional for Dirac-harmonic maps with curvature term is invariant under conformal transformations such that in this case the solutions of the resulting Euler–Lagrange equations share nice properties, one of them being the possibility of removing isolated point singularities if a certain energy is finite [7].

Note that the smoothness of weak solutions of (5.1), (5.2) can also be established with the help of Theorem 4.4, where we would consider the case $s=4$.

However, it is not hard to realize that the action functional for Dirac-harmonic maps with curvature term is still unbounded from below, meaning that the curvature term does not help in curing the analytic problems of the Dirac-harmonic map action functional.

### A potential from supersymmetric quantum field theory

As already explained in the Introduction, the action functionals of quantum field theory are built by the requirement that they are invariant under certain symmetry operations. It was shown in [2], see also [18,  Theorem 3.82], that the following potential

$$\begin{aligned} V_2(\phi ,\psi )=\frac{1}{2}|\nabla W(\phi )|^2+\frac{1}{2}{{\text {Hess}}~}W(\phi )(\psi ,\psi ) -\frac{1}{12}\langle R^N(\phi )(\psi ,\psi )\psi ,\psi \rangle \end{aligned}$$

preserves the invariance under supersymmetry transformations, making it a distinct potential for the supersymmetric nonlinear sigma model. Here, $W:N\rightarrow \mathbb {R}$ is a smooth function. On the other hand, it is not hard to realize that this particular potential does not preserve the invariance under conformal transformations on a two-dimensional domain manifold.

The resulting Euler–Lagrange equations acquire the following form:5.35.4In the case of a compact target $N$, the potential $V_2(\phi ,\psi )$ satisfies the following growth conditions:

$$\begin{aligned} |V_2(\phi ,\psi )|&\le C_1+C_2|\psi |^2+C_3|\psi |^4,\\ |V_{2\phi }(\phi ,\psi )|&\le C_4+C_5|\psi |^2+C_6|\psi |^4,\\ |V_{2\psi }(\phi ,\psi )|&\le C_7|\psi |+C_8|\psi |^3 \end{aligned}$$

for positive constants $C_i,i=1,\ldots 8$. In order to apply our main regularity result, that is Theorem 4.4, we need to satisfy the assumptions of case (1) with $s=2$ and $s=4$. Hence, a weak solution $(\phi ,\psi )\in \chi _t(M,N)$ with $t=4$ is smooth if the smallness condition (4.4) holds.

On the other hand, it can again easily be checked that the potential $V_2(\phi ,\psi )$ will not be helpful in achieving the positivity of the action functional for Dirac-harmonic maps or the positivity of the second variation although it respects the invariance under supersymmetry transformations in quantum field theory.

### Some further potentials

Let us give a short overview on some further potentials that fit into the framework of this article.

1. In quantum field theory, one often includes a term in the action functional that models the mass of a fermion. From a mathematical point of view, this is reflected in considering a potential of the form
  $$\begin{aligned} V_3(\phi ,\psi )=\frac{\lambda }{2}|\psi |^2, \qquad \lambda >0. \end{aligned}$$
  However, this particular potential is again not helpful to achieve a lower bound on the action functional (1.1). In addition, including the mass term destroys the conformal invariance of the action functional. The regularity of Dirac-harmonic maps coupled to a similar potential was studied in [41], where the Ricci curvature of the target manifold is used to define a potential that is quadratic in the spinors. The resulting Euler–Lagrange equations are 5.55.6 Note that the second equation states that we should find an eigenspinor of the twisted Dirac operator  with positive eigenvalue $\lambda$. The potential $V_3(\phi ,\psi )$ satisfies the following growth conditions:
  $$\begin{aligned} |V_3(\phi ,\psi )|&\le C_1|\psi |^2,\\ |V_{3\psi }(\phi ,\psi )|&\le C_2|\psi | \end{aligned}$$
  for positive constants $C_i,i=1,2$. To apply Theorem 4.4 again, we need to satisfy the assumptions of case (1) with $s=2$. Thus, as for the previous potential, a weak solution $(\phi ,\psi )\in \chi _t(M,N)$ with $t=4$ is smooth if the smallness condition (4.4) holds. On the other hand, we realize again that this specific potential cannot remove the indefinite character of the action functional for Dirac-harmonic maps.
2. From a purely mathematical perspective, a potential of the form
  $$\begin{aligned} V_4(\phi ,\psi )=V(\phi )-\exp (|\psi |^2) \end{aligned}$$
  could be beneficial. This particular potential will not respect the conformal invariance on a two-dimensional domain, and also the regularity theory established in Theorem 4.4 does not apply in this case. On the other hand, the potential $V_4(\phi ,\psi )$ may be helpful in order to establish the positivity of the action functional (1.1) and also its second variation (2.4).
  Inserting $V_4(\phi ,\psi )$ into the condition for the positivity of the action functional (3.1), we obtain
  $$\begin{aligned} \sum _{J=-\infty }^\infty |\alpha _J|^2\lambda _J-2\int _MV(\phi )\text {dvol}_g+2\int _M\exp (|\psi |^2)\text {dvol}_g\ge 0. \end{aligned}$$
  Although the potential $V_4(\phi ,\psi )$ contains an exponential factor, we still cannot ensure that it can compensate the negative eigenvalues of the Dirac term.
  Moreover, inserting $V_4(\phi ,\psi )$ into the conditions for the second variation to be positive, which are (3.2) and (3.3), we obtain the requirements
  $$\begin{aligned} -\iota (\eta ,\eta )\big (V_{\phi \phi }(\phi )\big )\ge C|\eta |^2e(\phi ,\psi ) \end{aligned}$$
  and  While the first condition can be satisfied on a non-compact target manifold $N$ if $e(\phi ,\psi )$ is finite, we still may encounter problems in controlling the Dirac term in the second inequality.
3. In the case of a vanishing spinor, that is $\psi =0$, the action functional (1.1) reduces to the one for harmonic maps with potential introduced in [19].
  The corresponding Euler–Lagrange equation is
  5.7 $$\begin{aligned} \tau (\phi )=-V_\phi (\phi ). \end{aligned}$$
  For harmonic maps with potential, we obtain the following variant of Theorem 4.4 generalizing [10,  Theorem 1.1] to higher dimensions:

#### Theorem 5.1

Let $m\ge 2$ and suppose that the potential $V(\phi )$ is smooth. Then, there exists $\epsilon >0$ depending on $(M,g), (N,h)$ such that if $\phi \in W^{1,2}(M,N)$ is a weak solution of (5.7) satisfying

5.8 $$\begin{aligned} \Vert \mathrm{d}\phi \Vert _{M^{2,2}(U)}\le \epsilon \end{aligned}$$

for some open subset $U\subset M$, then $\phi$ is smooth in $U$.

#### Proof

This follows directly from Theorem 4.8 and a standard bootstrap. $\square$

## Data Availability

Data sharing is not applicable to this article as no datasets were generated or analyzed during the current study.
